# Gemcitabine and Selected mTOR Inhibitors in Uterine Sarcomas and Carcinosarcoma Cells- an Isobolographic Analysis

**DOI:** 10.7150/ijms.48187

**Published:** 2020-10-18

**Authors:** Marcin Bobiński, Karolina Okła, Jarogniew Łuszczki, Wiesława Bednarek, Anna Wawruszak, Gema Moreno-Bueno, Pablo Garcia-Sanz, Magdalena Dmoszyńska-Graniczka, Rafał Tarkowski, Jan Kotarski

**Affiliations:** 1 Medical University of Lublin, I Chair and Department of Gynaecological Oncology and Gynaecology, Poland; 2 Medical University of Lublin, Chair and Department of Pathophisiology, Poland; 3 Medical University of Lublin, Chair and Department of Biochemistry and Molecular Biology, Poland; 4 MD Anderson Cancer Centre Madrid, Laboratorio de Investigación Traslacional Madrid, Spain

**Keywords:** uterine sarcomas, carcinosarcomas, mTOR, mTOR inhibitors, gemcitabine, isobolography, rapamycin

## Abstract

**Introduction:** mTOR inhibitors are anticancer agents affecting mTOR/AKT/PI3K pathway that is one of the most important in human cancer cells. Hyperactivation of mTOR/AKT/PI3K and overexpression of this pathway members are frequently reported in uterine sarcoma and carcinosarcoma.

Present study is aimed to assess the activity of the two mTOR inhibitors (rapamycin - RAP and sapanisertib - MLN) as a single agent and combined with gemcitabine (GEM, one of substances commonly used in systemic anticancer treatment) in uterine sarcoma and carcinosarcoma *in vitro* models.

**Material and methods:** SK-UT-1 and SK-UT1-B (uterine carcinosarcoma), MES-SA (leiomyosarcoma) and ESS-1 (endometrial stromal sarcoma) cell lines were used. An MTT assay was performed to examine the cytotoxicity of RAP, MLN and mixtures: RAP+MLN, RAP+GEM, MLN+GEM against these cells. The interactions between tested compounds were assessed in isobolographic analysis.

**Results and conclusions:** Carcinosarcoma cell lines (both SK-UT-1 and SK-UT-1B) do not respond to RAP and respond relatively weakly to MLN treatment. Additive and supraadditive effects were noted for combined treatment with GEM and MLN. Endometrial stromal sarcoma cell line (ESS-1) occured to be sensitive to both RAP and MLN, but the response was stronger for MLN. Additive effect of all tested drug combinations was observed for ESS-1. Leiomyosarcoma cell line (MES-SA) was found sensitive to both mTOR inhibitors. Additive effects in combinations of GEM, RAP and MLN were observed, what makes them promising for future preclinical and clinical trials. Additivity with slight tendency towards antagonism between GEM and MLN observed in MES-SA cell line is unexpected finding and might prompt the mechanistic research aimed to explain this phenomenon.

## Introduction

mTOR/AKT/PI3K is one of the most important signal transduction pathway in human cancer cells. It integrates many cell functions including growth, proliferation, translation and transcription 1.

Such properties made it one of the most actively investigated molecular target in oncology. Many substances affecting mTOR have been identified so far.

The mTOR protein executes its metabolic function by composing complexes with raptor and mLST8/GβL(TORC1) and with rictor and mSIN,GβL (TORC2) 1, 2.

TORC1 regulates mostly protein synthesis by phosphorylation of translation factors, TORC2 controls cell survival and cytoskeleton functions, both complexes are linked by AKT 3.

The most 'classic' mTOR inhibitor is rapamycin (sirolimus), from which the names of protein and pathway were created, it is natural compound isolated from culture of *Streptomyces hygroscopicus* bacteria. It is widely used as immunosupresant in prevention of transplanted organ rejection and restenosis of coronary vessels (stents coated with rapamycin). Rapamycin binds and deactivates mTOR molecule. It was proved to efficiently inhibit activity of TORC1 complex 4. The disadvantage of this mechanism from clinical point of view is quick feedback activation of AKT as a response for TORC1 inhibition 5. AKT has ability to activate TORC2 complex which, in simplification, 'substitutes' the metabolic function of TORC1 allowing the pathway to remain active 4. Due to this limitation, rapamycin is not widely utilized in oncology. Rapid development of resistance to rapamycin in cancer cells accelerated efforts to introduce novel generations of mTOR inhibitors.

MLN (also known as MLN 0128, INK128, TAK-228 and sapanisertib) is novel allosteric and ATP-competitive mTOR inhibitor. Unlike rapamycin, this compound inhibits both TORC1 and TORC2 complexes. The mechanism of overcoming rapamycin resistance by MLN was revealed by Rodik-Outmezguine et al. 4 Further studies confirmedactivity of this substance in i.a. pancreatic and bladder cancer cell lines and xenografts 6,7. At present, MLN undergoes clinical trials in many indications, including solid tumours. It is also under investigation as single agent in locally advanced or recurrent soft tissue sarcomas, the results are awaiting in 2020/2021 8.

Gemcitabine is commonly using chemotherapeutic, according to recent recommendations is one of agents to be used in standard systemic treatment of uterine sarcomas 9. Gemcitabine is one of the most active agents in this group of cancers although the obtained results remainunsatisfactory 10.

Hyperactivation of mTOR/AKT/PI3K and overexpression of this pathway members are frequently reported in uterine sarcoma and carcinosarcoma 5,11. Although, this pathway is considered to be one of most promising target for future therapies 5, 9, 12, none of tested mTOR inhibitors was approved by FDA to be used in treatment of uterine sarcomas and carcinosarcomas. Small number of studies on sarcomas and their unsatisfactory results are the reason for this situation. In most cases patients experienced limited clinical benefit accompanying by severe side effects due to toxicity of agents even in therapeutic doses 5, 13, 14.

The majority of studies that investigated the effectiveness of various mTOR inhibitors (both preclinical and clinical), in sarcomas, were designed to assess single agent activity 13, 14. RAP was previously tested in combination with GEM in clinical trial, but it gave inconclusive results and recuited only 4 patients with gynaecological sarcomas, so the efficacy and safety of this regimen still remain unknown 15.

There is limited number of studies already finished and ongoing assessing the performance of MLN added to conventional chemotherapy but these do not recruit patients with neither uterine sarcomas nor carcinosarcomas 16, 17. Furthermore there are no available data of efficacy of MLN in combination with gemcitabine.

Presently, there is a gap in knowledge about interactions between tested compounds in uterine sarcoma models. In present study we propose to evaluate the activity of both mentioned mTOR inhibitors in combination with gemcitabine as a standard therapeutic agent, that may indicate the directions for future therapeutic regimens.

The study is aimed to assess the activity of RAP and MLN as a single agent, in combination and combined with gemcitabine in uterine sarcomas and carcinosarcomas in vitro models.

## Material and methods

### Cell lines

Four cell lines were selected to experiments:

- SK-UT-1 and SK-UT1-B- derived from uterine carcinosarcoma (these cell lines represent two cellular populations of carcinosarcoma: sarcomatous and carcinomatous, respectively)

- MES-SA- derived from leiomyosarcoma

- ESS-1- derived from low grade endometrial stromal sarcoma.

Cell lines were obtained from the Laboratorio de Investigación Traslacional, MD Anderson Cancer Centre in Madrid, Spain. The SK-UT-1, SK-UT-1B and MES-SA cells were cultured according to ATCC recommendations 18. Due to the lack of ATCC recommendations for ESS-1 the culture was carried as described by Gunawan et al 19. Human skin fibroblasts (HSF) cell line was used to assess the impact of tested compounds on normal cells. HSF was developed in Medical University of Lublin from young volunteer skin samples. Fibroblasts were isolated from small pieces of human skin that were suspendend to the bottom of wells of 24-multiwell plate and cultured in medium (DMEM/RPMI, (Sigma‐Aldrich, USA and PAN-Biotech,Germany, respectively) 1:1 mixture containing 10% of FBS (PAN-Biotech, Germany) and 1% penicillin-streptomycin (Sigma‐Aldrich, USA)) supplemented with fibroblast growth factor. After about 2-weeks of incubation the monolayer cell line was obtained, according to the method described elsewere 20

The methodology of cell cultures and detailed description of used cell lines was described in details previously 21, 22.

### Cell viability assay

Cell viability was assessed by performing MTT test. Cells were plated in microplates and incubated in presence of various concentrations of RAP (Sigma‐Aldrich, USA), MLN (Cayman Chemical USA), GEM (EBEWE Pharma, Austria) and following 1:1 mixtures: RAP+MLN, RAP+GEM, MLN+GEM for 96 h. Afterwards MTT (Sigma‐Aldrich, USA) (3-(4,5-dimethylthiazol-2-yl)-2,5-diphenyltetrazolium bromide) solution was added for 3 h of incubation. Living cells metabolize MTT into purple formazan crystals that are solubilised. The solution was examined optically in light 570 nm length with ELX-800 plate reader (Bio-Tek Instruments, USA) and analyzed with Gen5 software (Bio-Tek Instruments, USA). All experiments were repeated three times and the median value was used for calculations.

### Statistical analysis and isobolography

The IC50 values were calculated by computer-assisted log-probit analysis. Differences in cells viability between control and particular concentrations of tested compounds were evaluated with one-way ANOVA Tukey's post-hoc testing. P<0.05 was considered to indicate a statistically significant difference. The analysis was performed using GraphPad Prism 5.0 (GraphPad Software, USA).

In order to analyze pharmacodynamic interactions between tested substances isoblography was used. The methodology of this analysis was described in our previous paper 22. Briefly, isobolography is statistical method designed to determine the type of interactions between compounds, active against particular cell line. It is based on results of cell viability tests performed in cells treated with both single agents and mixtures. The results of such analysis are presented as isobolograms indicating type of interactions (defined as: additivity, supra-additivity or antagonism). Detailed description of mathematical background of isobolographic analysis was presented by Tallarida and Luszczki 23,24. The analysis of our results was performed according to their methodology.

## Results

### Single agent treatment

**Figure 1:**
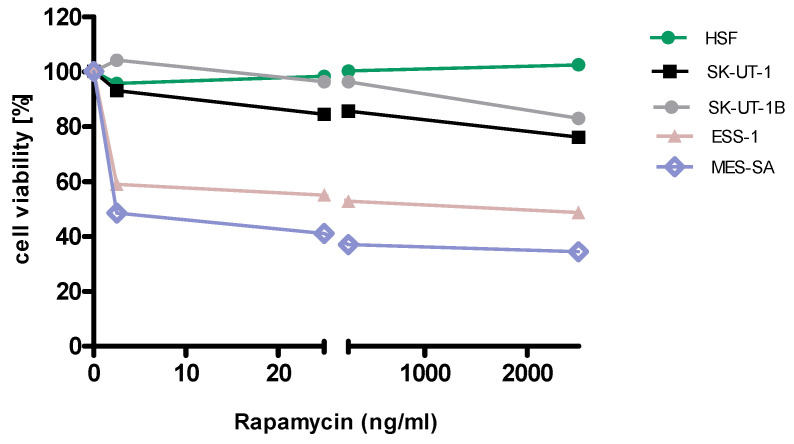
Anti-proliferative effects of rapamycin on the cell lines

**Figure 2:**
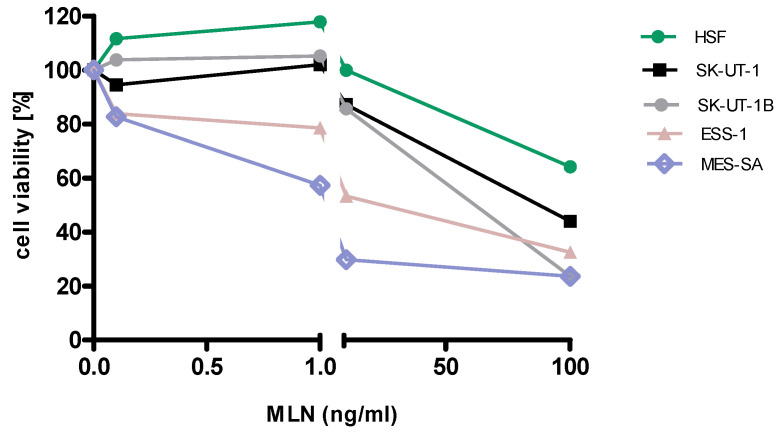
Anti-proliferative effects of MLN on the cell lines

Detailed results of single agent activity of RAP and MLN measured in MTT test in SK-UT-1, SK-UT-1B, ESS-1 and MES-SA cell lines are presented on Figures 1 and 2. The single agent activity of gemcitabine in tested cell lines was previously reported by our research group 22.

IC_50_ values obtained for each agent in monotherapy in all tested cell lines are summarized in table 1. Due to weak response to RAP in SK-UT-1 and SK-UT-1B cell lines IC_50_ values were not determined.

**Figure 3:**
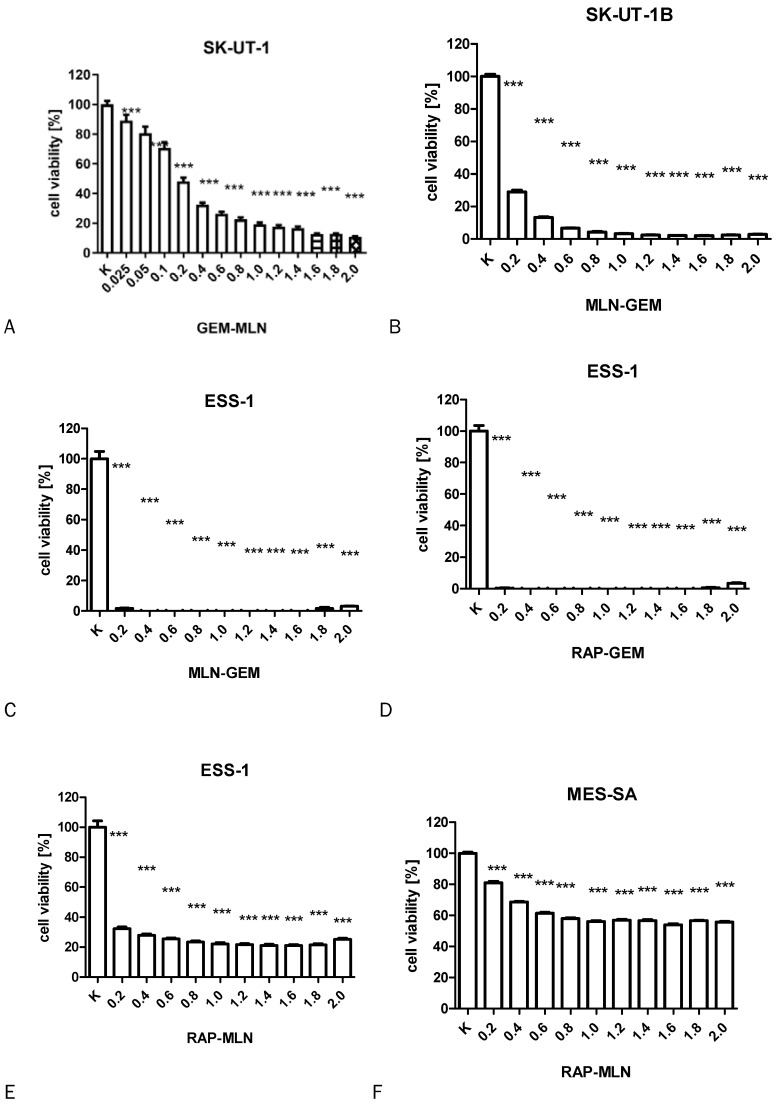

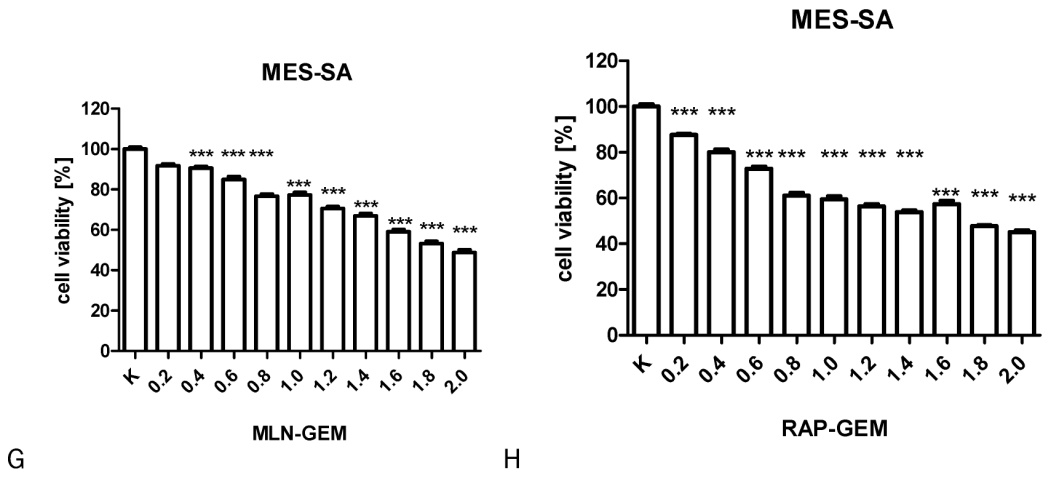
The influence of RAP, GEM and MLN combinations on SK-UT-1 [A], SK-UT1B [B], ESS-1 [C,D,E] and MES-SA [F,G,H](***p<0.001). The values on axis X represent the multiplicity of calculated IC_50_.

The results of MTT test performed to assess the activity of combinations of tested drugs in SK-UT-1, SK-UT-1B, ESS-1 and MES-SA were presented in Figure 3. The lack of response to RAP of both carcinosarcoma-derived cell lines implicated with inability to assess the activity of its combination with other drugs in these lines, so the results were obtained only for GEM and MLN.

### Isobolographic analysis

**Figure 4:**
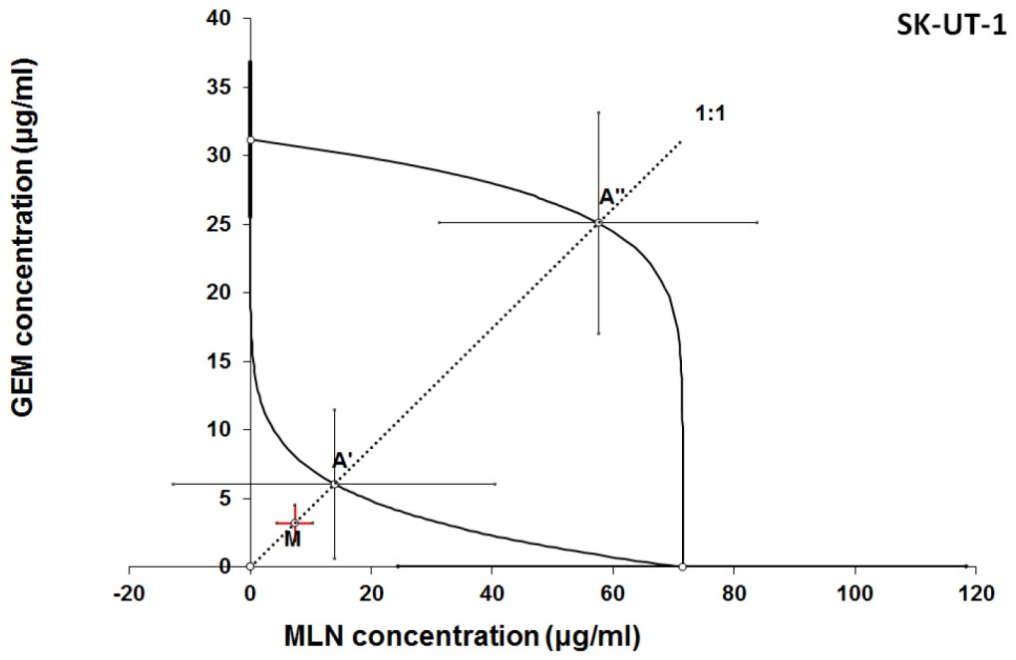
** Isobologram** showing interaction between gemcitabine (GEM) and MLN0128 (MLN) with respect to their anti-proliferative effects in the cancer cell line (SK-UT-1) measured *in vitro* by the MTT assay. The median inhibitory concentrations (IC_50_) for GEM and MLN are plotted graphically on the X- and Y-axes, respectively. The solid lines on the X and Y axes represent the S.E.M. for the IC_50_ values for the studied drugs administered alone. The lower and upper isoboles of additivity represent the curves connecting the IC_50_ values for GEM and MLN administered alone. The dotted line starting from the point (0, 0) corresponds to the fixed-ratio of 1:1 for the combination of GEM with MLN. The points A' and A” depict the theoretically calculated IC_50 add_ values for both, lower and upper isoboles of additivity. The point M represents the experimentally-derived IC_50 mix_ value for total dose of the mixture expressed as proportions of GEM and MLN that produced a 50% anti-proliferative effect (50% isobole) in the cancer cell line (SK-UT-1) measured *in vitro* by the MTT assay. On the graph, the S.E.M. values are presented as horizontal and vertical error bars for every IC_50_ value. Although the experimentally-derived IC_50 mix_ value is placed below the point A', the interaction between GEM and MLN for the cancer cell line SK-UT-1 is additive.

**Figure 5:**
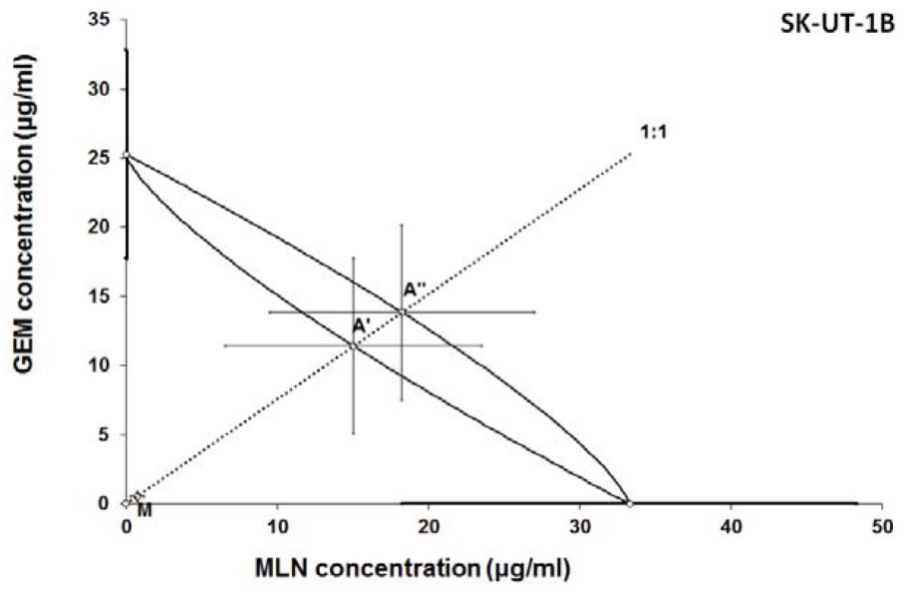
Isobologram showing interaction between gemcitabine (GEM) and MLN0128 (MLN) with respect to their anti-proliferative effects in the cancer cell line (SK-UT-1B) measured *in vitro* by the MTT assay. The median inhibitory concentrations (IC_50_) for GEM and MLN are plotted graphically on the X- and Y-axes, respectively. The solid lines on the X and Y axes represent the S.E.M. for the IC_50_ values for the studied drugs administered alone. The lower and upper isoboles of additivity represent the curves connecting the IC_50_ values for GEM and MLN administered alone. The dotted line starting from the point (0, 0) corresponds to the fixed-ratio of 1:1 for the combination of GEM with MLN. The points A' and A” depict the theoretically calculated IC_50 add_ values for both, lower and upper isoboles of additivity. The point M represents the experimentally-derived IC_50 mix_ value for total dose of the mixture expressed as proportions of GEM and MLN that produced a 50% anti-proliferative effect (50% isobole) in the cancer cell line (SK-UT-1B) measured *in vitro* by the MTT assay. On the graph, the S.E.M. values are presented as horizontal and vertical error bars for every IC_50_ value. Because the experimentally-derived IC_50 mix_ value is placed significantly below the point A', the interaction between GEM and MLN for the cancer cell line SK-UT-1B is supra-additive (synergistic). ***P<0.001 vs the respective IC_50 add_ values.

**Figure 6:**
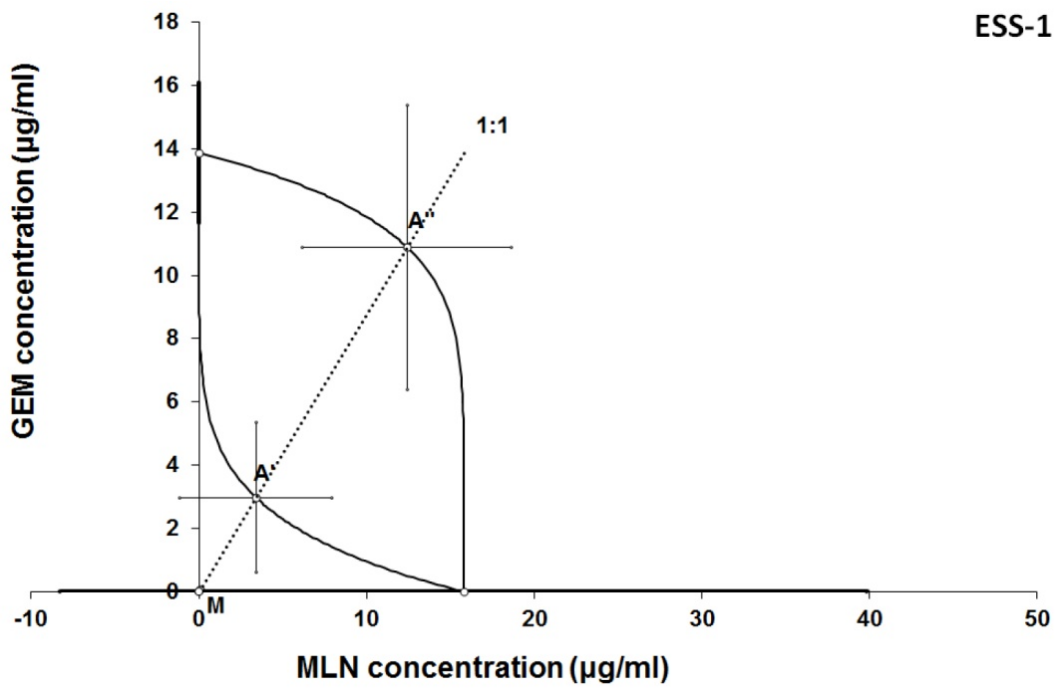
Isobologram showing interaction between GEM andbMLN with respect to their anti-proliferative effects in the cancer cell line (ESS-1) measured *in vitro* by the MTT assay. The median inhibitory concentrations (IC_50_) for GEM and MLN are plotted graphically on the X- and Y-axes, respectively. The solid lines on the X and Y axes represent the S.E.M. for the IC_50_ values for the studied drugs administered alone. The lower and upper isoboles of additivity represent the curves connecting the IC_50_ values for GEM and MLN administered alone. The dotted line starting from the point (0, 0) corresponds to the fixed-ratio of 1:1 for the combination of GEM with MLN. The points A' and A” depict the theoretically calculated IC_50 add_ values for both, lower and upper isoboles of additivity. The point M represents the experimentally-derived IC_50 mix_ value for total dose of the mixture expressed as proportions of GEM and MLN that produced a 50% anti-proliferative effect (50% isobole) in the cancer cell line (ESS-1) measured *in vitro* by the MTT assay. On the graph, the S.E.M. values are presented as horizontal and vertical error bars for every IC_50_ value. Although the experimentally-derived IC_50 mix_ value is placed below the point A', the interaction between GEM and MLN for the cancer cell line ESS-1 is additive.

**Figure 7:**
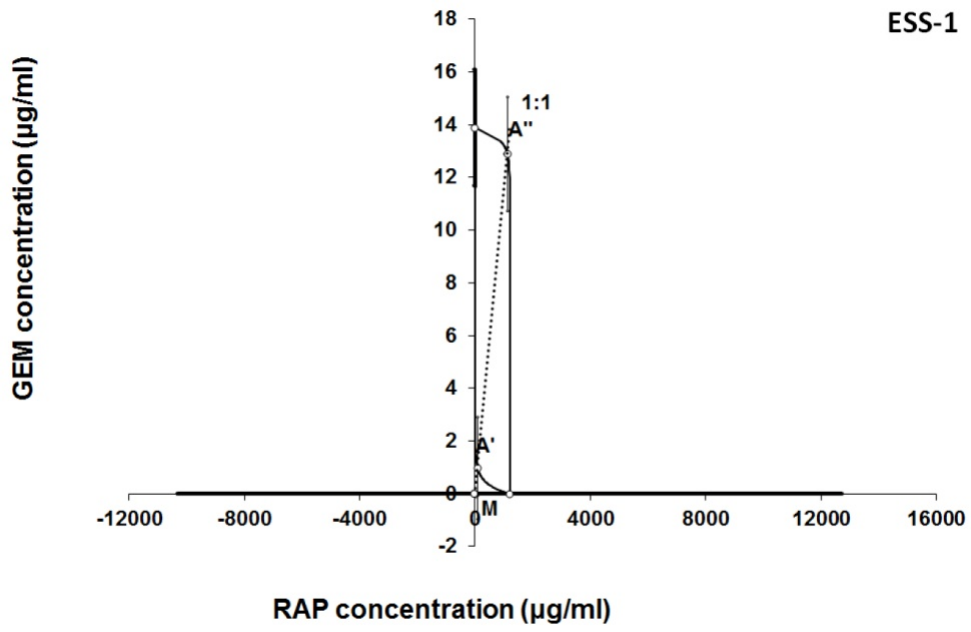
Isobologram showing interaction between gemcitabine (GEM) and rapamycine (RAP) with respect to their anti-proliferative effects in the cancer cell line (ESS-1) measured *in vitro* by the MTT assay. The median inhibitory concentrations (IC_50_) for GEM and RAP are plotted graphically on the X- and Y-axes, respectively. The solid lines on the X and Y axes represent the S.E.M. for the IC_50_ values for the studied drugs administered alone. The lower and upper isoboles of additivity represent the curves connecting the IC_50_ values for GEM and RAP administered alone. The dotted line starting from the point (0, 0) corresponds to the fixed-ratio of 1:1 for the combination of GEM with RAP. The points A' and A” depict the theoretically calculated IC_50 add_ values for both, lower and upper isoboles of additivity. The point M represents the experimentally-derived IC_50 mix_ value for total dose of the mixture expressed as proportions of GEM and RAP that produced a 50% anti-proliferative effect (50% isobole) in the cancer cell line (ESS-1) measured *in vitro* by the MTT assay. On the graph, the S.E.M. values are presented as horizontal and vertical error bars for every IC_50_ value. Although the experimentally-derived IC_50 mix_ value is placed below the point A', the interaction between GEM and RAP for the cancer cell line ESS-1 is additive.

**Figure 8:**
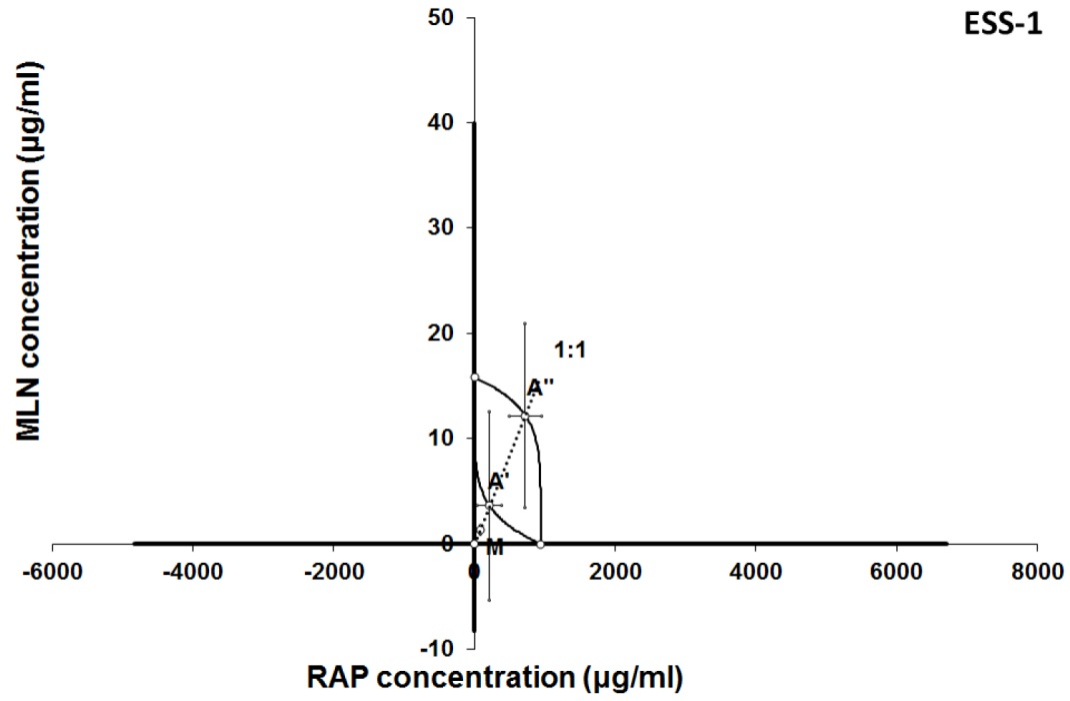
Isobologram showing interaction between MLN0128 (MLN) and rapamycine (RAP) with respect to their anti-proliferative effects in the cancer cell line (ESS-1) measured *in vitro* by the MTT assay. The median inhibitory concentrations (IC_50_) for MLN and RAP are plotted graphically on the X- and Y-axes, respectively. The solid lines on the X and Y axes represent the S.E.M. for the IC_50_ values for the studied drugs administered alone. The lower and upper isoboles of additivity represent the curves connecting the IC_50_ values for MLN and RAP administered alone. The dotted line starting from the point (0, 0) corresponds to the fixed-ratio of 1:1 for the combination of MLN with RAP. The points A' and A” depict the theoretically calculated IC_50 add_ values for both, lower and upper isoboles of additivity. The point M represents the experimentally-derived IC_50 mix_ value for total dose of the mixture expressed as proportions of MLN and RAP that produced a 50% anti-proliferative effect (50% isobole) in the cancer cell line (ESS-1) measured *in vitro* by the MTT assay. On the graph, the S.E.M. values are presented as horizontal and vertical error bars for every IC_50_ value. Although the experimentally-derived IC_50 mix_ value is placed below the point A', the interaction between MLN and RAP for the cancer cell line ESS-1 is additive.

**Figure 9:**
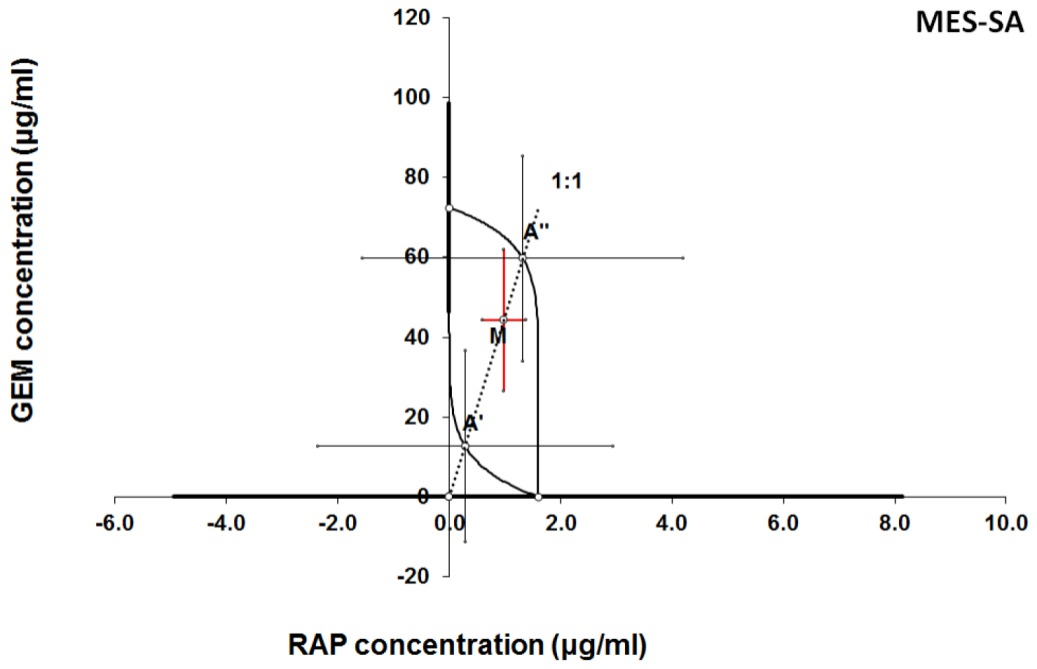
Isobologram showing interaction between gemcitabine (GEM) and rapamycine (RAP) with respect to their anti-proliferative effects in the cancer cell line (MES-SA) measured *in vitro* by the MTT assay. The median inhibitory concentrations (IC_50_) for GEM and RAP are plotted graphically on the X- and Y-axes, respectively. The solid lines on the X and Y axes represent the S.E.M. for the IC_50_ values for the studied drugs administered alone. The lower and upper isoboles of additivity represent the curves connecting the IC_50_ values for GEM and RAP administered alone. The dotted line starting from the point (0, 0) corresponds to the fixed-ratio of 1:1 for the combination of GEM with RAP. The points A' and A” depict the theoretically calculated IC_50 add_ values for both, lower and upper isoboles of additivity. The point M represents the experimentally-derived IC_50 mix_ value for total dose of the mixture expressed as proportions of GEM and RAP that produced a 50% anti-proliferative effect (50% isobole) in the cancer cell line (MES-SA) measured *in vitro* by the MTT assay. On the graph, the S.E.M. values are presented as horizontal and vertical error bars for every IC_50_ value. The experimentally-derived IC_50 mix_ value is placed between the points A' and A” and thus, the interaction between GEM and RAP for the cancer cell line MES-SA is additive.

**Figure 10:**
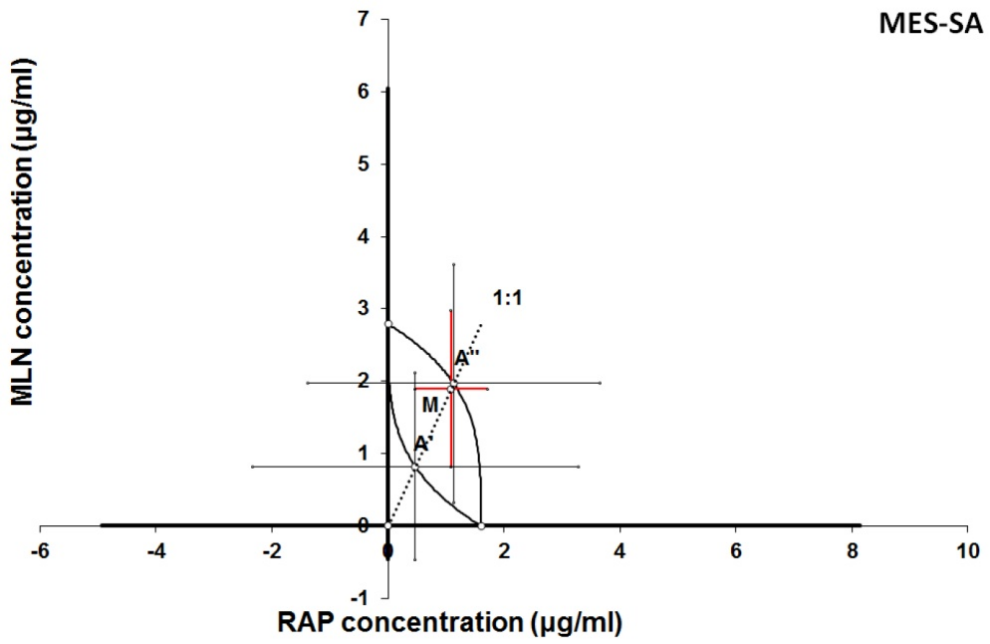
Isobologram showing interaction between MLN0128 (MLN) and rapamycine (RAP) with respect to their anti-proliferative effects in the cancer cell line (MES-SA) measured *in vitro* by the MTT assay. The median inhibitory concentrations (IC_50_) for MLN and RAP are plotted graphically on the X- and Y-axes, respectively. The solid lines on the X and Y axes represent the S.E.M. for the IC_50_ values for the studied drugs administered alone. The lower and upper isoboles of additivity represent the curves connecting the IC_50_ values for MLN and RAP administered alone. The dotted line starting from the point (0, 0) corresponds to the fixed-ratio of 1:1 for the combination of MLN with RAP. The points A' and A” depict the theoretically calculated IC_50 add_ values for both, lower and upper isoboles of additivity. The point M represents the experimentally-derived IC_50 mix_ value for total dose of the mixture expressed as proportions of MLN and RAP that produced a 50% anti-proliferative effect (50% isobole) in the cancer cell line (MES-SA) measured *in vitro* by the MTT assay. On the graph, the S.E.M. values are presented as horizontal and vertical error bars for every IC_50_ value. The experimentally-derived IC_50 mix_ value is placed close to the point A” and thus, the interaction between MLN and RAP for the cancer cell line MES-SA is additive.

**Figure 11:**
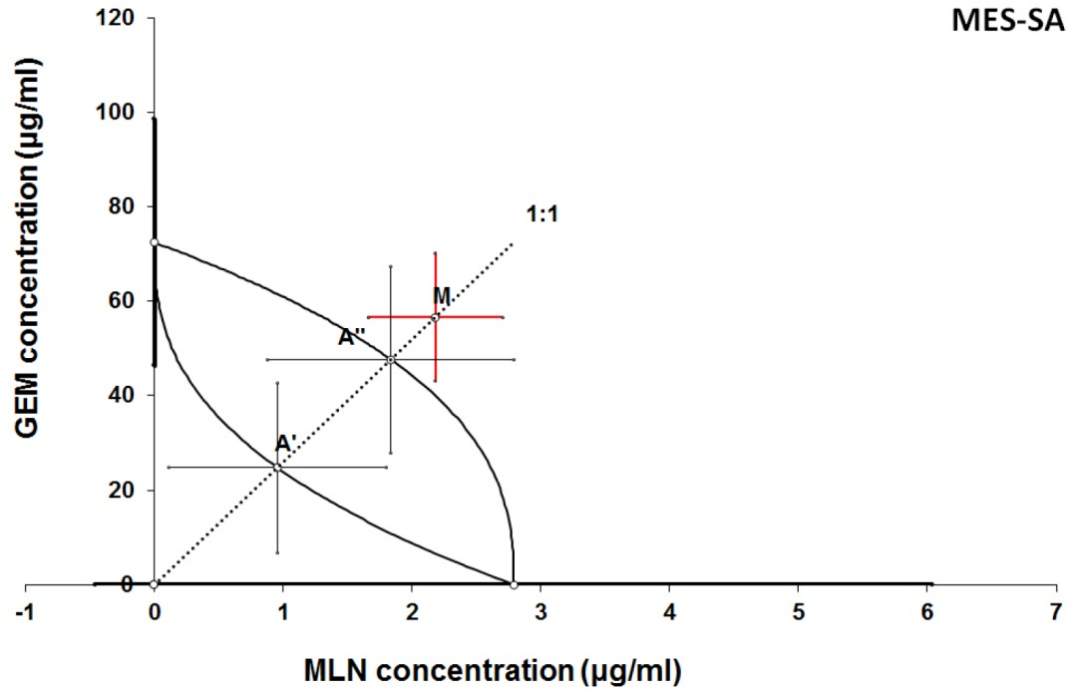
Isobologram showing interaction between GEM and MLN with respect to their anti-proliferative effects in the cancer cell line (MES-SA) measured *in vitro* by the MTT assay. The median inhibitory concentrations (IC_50_) for GEM and MLN are plotted graphically on the X- and Y-axes, respectively. The solid lines on the X and Y axes represent the S.E.M. for the IC_50_ values for the studied drugs administered alone. The lower and upper isoboles of additivity represent the curves connecting the IC_50_ values for GEM and MLN administered alone. The dotted line starting from the point (0, 0) corresponds to the fixed-ratio of 1:1 for the combination of GEM with MLN. The points A' and A” depict the theoretically calculated IC_50 add_ values for both, lower and upper isoboles of additivity. The point M represents the experimentally-derived IC_50 mix_ value for total dose of the mixture expressed as proportions of GEM and MLN that produced a 50% anti-proliferative effect (50% isobole) in the cancer cell line (MES-SA) measured *in vitro* by the MTT assay. On the graph, the S.E.M. values are presented as horizontal and vertical error bars for every IC_50_ value. The experimentally-derived IC_50 mix_ value is placed above the point A”, indicating a tendency towards antagonism between GEM and MLN for the cancer cell line MES-SA.

The results of isobolographic analysis indicating type of interactions between examined substances are presented in Figures 4-11. In SK-UT-1 cell line additivity of GEM+MLN was observed, while in SK-UT-1B cell line supr-additive (synergistic) interaction was noted. GEM+MLN combination showed additivity also in ESS-1, in this cell line addition was also deteted in combinations: GEM+RAP and MLN+RAP. Additivity was observed for MLN+RAP and GAM+RAP. Interestingly for combination GEM+MLN in MES-SA cell line additivity with slight (not statistically significant) tendency to antagonism was observed.

## Discussion

Resistance of carcinosarcoma cell lines (SK-UT-1, SK-UT-1B) to rapamycin and relatively weaker response to MLN than observed in other investigated cell lines was noticed. Such observation were not reported yet in available literature. The resistance to rapamycin might be explained by feedback activation of AKT and as consequence activation of mTOR pathway. The results of genetic analysis by Jones et al. revealed that the activity of mTOR pathway in carcinosarcomas is less altered, comparing to type I endometrial cancer 25. There are no reliable data regarding the role of mTOR pathway in this type of malignancies. On the other hand relative weak response to MLN suggests that this pathway may not play a pivotal role in this type of tumour, but this issue requires further investigation.

Interestingly, we noticed additive and supra-additive effects of combined GEM and MLN in SK-UT-1 and SK-UT-1B. Taking into consideration that we achieved relatively deep inhibition of cell viability in presence of relatively low concentrations of tested compounds. When both substances were mixed in 1/10 of their IC_50_ cell viability was decreased to about 50% in SK-UT-1 and about 30% in SK-UT-1B such results have to be considered as promising and this combination might occur to be useful in clinical practice. No data regarding activity of mTOR inhibitors in combination with chemotherapy in uterine carcinosacoma models or in clinical practice are available in literature up to date. Bae‐Jump et al. revealed synergistic effect of rapamycin and cisplatin combination in endometrial cancer cells, however the cells that were used were derived from type I endometrial cancer 26. Taking into consideration differences in mTOR pathway alterations between type I endometrial carcinoma and carcinosarcoma, supported by observed in present study resistance of carcinosarcoma cell lines to rapamycin, therefore comparing this studies seems to be doubtful. On the other hand similarities between endometrial cancer and cancerous part of carcinosarcoma are well known and resulted with its qualification as type 2 endometrial cancer 27. So the results obtained especially in SK-UT-1B cell line allow to expect combination of GEM and MLN to be effective also in endometrial cancer.

Additive effect of all tested combinations was observed in endometrial stromal sarcoma cell line (ESS-1), though it responded to treatment with rapamycin moderately. Endometrial stromal sarcoma tumours are, in most cases, hormone-sensitive. In systemic treatment of these tumours anti-oestrogen agents are widely using in combinations with cytostatics and radiotherapy 9. Although, especially in cases of recurrence, hormonal resistance may occur 28. There are reports suggesting that the mechanism of acquiring hormone resistance may be related with mTOR pathway 29. As it was mentioned above the number of papers regarding mTOR inhibitors in sarcomas is very limited. The role of mTOR inhibitors in overcoming hormone-insensivity of ESS was discussed by Martin-Liberal at al., they presented case of successful reverse of hormone-insensivity by mTOR inhibitor treatment in patient suffering from metastatic ESS 28. Such observations justify efforts to investigate the efficacy of mTOR inhibitors for hormone-dependent cancers. The additivity observed between tested compounds may be used in future combinations with hormonal agents in clinical or preclinical trials giving a chance to combine three different mechanisms of activity against ESS:

- DNA synthesis alteration by GEM

- mTOR pathway inhibition

- Oestrogen receptor deactivation.

The importance of mTOR signalling in leiomyosarcoma tumours biology is known 5, 29. In clinical practice GEM is one of the most common cytostatic that patients suffering from are given 9. Surprisingly, in MES-SA we observed the weakest response to GEM among tested cell lines. On the other hand, we noted very strong response to treatment with RAP. Another interesting finding is the tendency to antagonistic relation between GEM and MLN. Nowadays mechanisms of this phenomenon are not recognized and require further explanation but even such observation on preclinical model may suggest possible limitation for future clinical trials. The rest of tested combinations gave much more optimistic results, we noted additive effect between both GEM-RAP and MLN-RAP. These observations suggest that such combinations may occur useful in clinical practice. Furthermore, the fact that GEM is widely accepted therapeutic option in uterine leiomyosarcoma may simplify designing of future clinical trials aimed to assess the efficacy of addition mTOR inhibitor to such standard treatment.

There are two main limitations of mTOR inhibitors usefulness in clinical practice. One is acquisition of resistance by cancer cells, second one is toxicity to patients. Acquiring the resistance to mTOR inhibitors (most commonly rapamycin and its derivates) was detected in various tumours' models *in vitro* as well as *in vivo* in patients suffering from such conditions as i.a. breast cancers, gliomas 3,4,31. The toxicity of mTOR inhibitors is widely known, the most commonly hematologic, gastrointestinal and metabolic side effects are observed. These side effects often lead to serious complication and are one of causes of treatment discontinuation 32.

Multidrug regimens have a chance to overcome both of them. Obviously, these may allow decreased dosing by exploiting additivity and synergy in drugs combinations. Hence, lead to reduce dose-depending toxicity, but targeting multiple cellular pathways at the same time gives, also a chance to avoid formation of cells resistance to anti-neoplastic agent. When many pathways are altered simultaneously then single mutation resulting with resistance to one of agents is not associated with survival handicap and risk of resistance is much lower comparing to monotherapy.

The mTOR pathway is considered as one of regulating mechanisms responsible for cell sensitivity to GEM. Such relation was already proved in various cancers including breast and pancreatic 33, 34,35. Yang et al. investigated the mechanism of resistance to GEM in breast cancer model, they noted hyperactivation of mTOR pathway in cells insensitive to treatment with GEM. Hence, they concluded that the resistance to GEM is mainly mediated by activation of the mTOR/AKT/PI3K signalling pathway. Interestingly they observed that inhibition of mTOR/AKT/PI3K sensitizes gemcitabine-resistant cells to GEM 35. Deeper insight in relations between mTOR pathway and GEM activity in cancer cells makes interpretation of obtained results even more complex. Mechanism described above easily explains additive and supradditive effects observed between GEM and both mTOR inhibitors in almost all of tested combinations. But in this light tendency to antagonism observed between GEM and MLN in MES-SA cell line still remains surprising, especially if we take into consideration known, important, role of mTOR pathway in metabolism of these tumours. In contrary the effect observed in combination GEM and RAP was additive. Such differences could be potentially explained by relatively weak response to GEM in monotherapy in this cell line. Furthermore such effect can be explained by differences in molecular activity between RAP and MLN in binding to TORC1 and TORC2 complexes, resulted with lower affinity to binding site of MLN than RAP, described in details by Rodrik-Outmezguine et al. 4.

Interestingly, the combination of GEM and mTOR inhibitors is already in early phases clinical trials. So far results of the studies are inconsistent. Combination of temsirolimus (analogue of RAP) and GEM showed promising activity in pancreatic cancer 30. Contrary, other analogue of RAP - everolimus combined with GEM/cisplatine show no clinical benefit in phase I/II study 35. The results obtained in present study support the rationale for planning further trials in this field.

The present study is not free of limitations. The most important is using only 2D models of uterine sarcomas and carcinosarcoma, such models are easily reproducible and allow to perform isobolographic analysis of relations between tested substances, but on the other hand do not fully reflect behaviour of tumours *in vivo*. Secondly, the reported project do not include any mechanistic research, partially the activity mechanisms of used compounds were previously described 4,5,5,7,26,29,30,33,35 but further studies aimed to describe mechanisms of cellular response to combined treatment are strongly indicated.

## Conclusions

1. Carcinosarcoma cell lines (both SK-UT-1 and SK-UT-1B) do not respond to RAP and respond relatively weak to MLN treatment. Although, additive and supraadditive effects were noted in combined treatment with GEM and MLN.

2. Additive effect in all tested combinations was observed in endometrial stromal sarcoma cell line (ESS-1). This suggests that combinations of mTOR inhibitors and GEM might be effective combination in treatment of such tumours, especially in cases of hormone-insensivity.

3. Leiomyosarcoma cell line (MES-SA) appeared as the most sensitive to both mTOR inhibitors among all of tested cell lines. Additive effects observed in combinations of GEM, RAP and MLN make them promising for future preclinical and clinical trials.

4. Tendency to antagonism between GEM and MLN observed in MES-SA cell line is unexpected finding and might prompt mechanistic research aimed to explain this phenomenon

## Figures and Tables

**Table 1:** Effects of rapamycin, MLN and gemcitabine on viability of SK-UT-1, SK-UT-1B, ESS-1 and MES-SA measured *in vitro* by the MTT assay. *IC50 values for GEM were presented in previous report 19.

Cell lines	Rapamycin (RAP) IC_50_ (ng/ml)	MLN 128 (MLN) IC_50_ (ng/ml)	Gemcitabine (GEM)* IC_50_ (ng/ml)
SK-UT-1	NA	214.2	31.173
SK-UT-1B	NA	33.260	25.243
ESS-1	971.505	15.808	13,875
MES-SA	1.602	2.789	72,482

## Abbreviations

AKT: Protein Kinase B
CS: carcinosarcoma
ESS: endometrial stromal sarcoma
FDA: U.S. Food and Drug Administration
GEM: gemcitabine
MLN: INK128 (also known as MLN 0128, TAK-228, sapanisertib)
LMS: leiomyosarcoma
mLST8/GβL: mammalian LST8/G-protein β-subunit like protein
mSIN1: mammalian stress-activated protein kinase interacting protein 1
mTOR: mammalian target of rapamycin kinase
MTT: 3-(4,5-dimethylthiazol-2-yl)-2,5-diphenyltetrazolium bromide
PI3K: phosphoinositide 3-kinases
RAP: rapamycin
Raptor: regulatory associated protein of mTOR
Rictor: rapamycin-insenstive companion of mTOR
